# Comparison between Flotrac-Vigileo and Bioreactance, a totally noninvasive method for cardiac output monitoring

**DOI:** 10.1186/cc7884

**Published:** 2009-05-19

**Authors:** Sophie Marqué, Alain Cariou, Jean-Daniel Chiche, Pierre Squara

**Affiliations:** 1 Medical Intensive Care Unit, Cochin Hospital, 27 rue du Faubourg Saint-Jacques 75679 Paris Cedex 14, France; 2 Paris Descartes University, Medical School, 15 rue de l'Ecole de Médecine 75270 Paris Cedex 06, France; 3 Clinique Ambroise Paré, 27 bd Victor Hugo, 92200 Neuilly-sur-Seine, France

## Abstract

**Introduction:**

This study was designed to compare the clinical acceptability of two cardiac output (CO) monitoring systems: a pulse wave contour-based system (FloTrac-Vigileo) and a bioreactance-based system (NICOM), using continuous thermodilution (PAC-CCO) as a reference method.

**Methods:**

Consecutive patients, requiring PAC-CCO monitoring following cardiac surgery, were also monitored by the two other devices. CO values obtained simultaneously by the three systems were recorded continuously on a minute-by-minute basis.

**Results:**

Continuous recording was performed on 29 patients, providing 12,099 simultaneous measurements for each device (417 ± 107 per patient). In stable conditions, correlations of NICOM and Vigileo with PAC-CCO were 0.77 and 0.69, respectively. The bias was -0.01 ± 0.84 for NICOM and -0.01 ± 0.81 for Vigileo (NS). NICOM relative error was less than 30% in 94% of the patients and less than 20% in 79% vs. 91% and 79% for the Vigileo, respectively (NS). The variability of measurements around the trend line (precision) was not different between the three methods: 8 ± 3%, 8 ± 4% and 8 ± 3% for PAC-CCO, NICOM and Vigileo, respectively. CO changes were 7.2 minutes faster with Vigileo and 6.9 minutes faster with NICOM (*P *< 0.05 both systems vs. PAC-CCO, NS). Amplitude of changes was not significantly different than thermodilution. Finally, the sensitivity and specificity for predicting significant CO changes were 0.91 and 0.95 respectively for the NICOM and 0.86 and 0.92 respectively for the Vigileo.

**Conclusions:**

This study showed that the NICOM and Vigileo devices have similar monitoring capabilities in post-operative cardiac surgery patients.

## Introduction

Until recently, continuous cardiac output (CO) monitoring required an invasive method, via a pulmonary artery catheter for thermodilution. During the past decade, several less invasive methods have been proposed [1,2]. Among these techniques, the FloTrac-Vigileo™ which uses arterial pressure signal monitoring to assess stroke volume, has given interesting preliminary results, but still requires an arterial catheterization [3]. A totally Non Invasive CO Monitoring (NICOM™) device, based on chest bioreactance, has been used in the majority of patients after cardiac surgery and could be useful in monitoring critically ill patients [4,5]. We designed this study to compare the clinical acceptability of the Vigileo™ and NICOM™ devices in critically ill patients, using semi-continuous thermodilution CO (PAC-CCO) as a reference method.

## Methods and materials

### Patients

We studied consecutive patients requiring PAC-CCO monitoring in the immediate postoperative period following pre-scheduled cardiac surgery. Patients were treated according to our standard protocols and no specific intervention was performed for this study. In each patient, a radial arterial catheter and a PAC-CCO catheter (Edwards Life Sciences, Irvine, CA, USA) were inserted preoperatively and maintained in place during the immediate postoperative period. The correct positioning of the PAC-CCO was checked by systematic chest x-rays at 1, 4, and 12 hours postoperatively. Postoperative echocardiography was systematically performed, to check for intracardiac shunts and significant tricuspid regurgitation.

The Vigileo™ (Edwards Lifesciences, Irvine, CA, USA) monitor with software version 1.01 was connected to the radial artery catheter via the FloTrac™ (Edwards Lifesciences, Irvine, CA, USA) pressure sensor. This recently introduced system calculates continuous CO on arterial pressure waveform characteristics but does not require external calibration. Individual demographic data, including height, weight, age, gender, and the real-time arterial pressure waveform analysis, are used to estimate arterial compliance.

The NICOM™ system (Cheetah Medical, Wilmington, DE, USA) requires the connection of four double electrode stickers placed on the thorax. Upper stickers were placed across the mid-left and right clavicles, and lower stickers were placed across the mid-left and right-last rib. In each electrode pair, the upper electrode delivers a small alternating current that has propagation characteristics that are sensed along the thorax by the lower electrode pairs, thus providing a measure of bioreactance (i.e., analysis of the variation in the frequency spectra of a delivered oscillating current that occurs when the current traverses the thoracic cavity, as opposed to the traditional bioimpedance, which relies only on analysis of changes in signal amplitude). This yields a signal-to-noise ratio that is about 100-fold greater than traditional bioimpedance [6].

### Data collection

For each patient, usual demographic data, type of surgery, and Simplified Acute Physiologic Score (SAPS) II were collected. Following intensive care unit (ICU) admission, CO values simultaneously furnished by the PAC-CCO, Vigileo™, and NICOM™ devices were automatically and almost continuously recorded using a computer data logger on a minute-by-minute basis. Periods of time in which one of the three devices gave unrealistic results for evident technical reasons were eliminated manually. For the reference method, one patient with severe tricuspid regurgitation was removed. NICOM™ disconnection is identified by the system and corresponding data were eliminated accordingly. For the Vigileo™, we eliminated periods of time where there was a loss of radial artery signal identified by a very low CO value equal or close to zero.

### Endpoints

Clinical acceptability was defined by four criteria for which we prospectively determined the tolerances as previously described [5]. In summary, the new technologies (Vigileo™ and NICOM™) were considered as: acceptably accurate when bias of measurement was less than 20%; acceptably precise when random error of measurements around the mean value were less than 20%; acceptably responsive when time delay and amplitude of change were at least equivalent to PAC-CCO, and acceptably reliable when sensibility and sensitivity in detecting simultaneous directional changes in CO was close to one. For this final criteria, unacceptable discordances in directional changes were defined as a difference of more than 20% between the two slopes or as a negative Intra Class Correlation (ICC). Our local institutional review board approved this protocol. Informed consent was obtained from each patient.

### Data analysis

Estimates were reported as mean ± standard deviation (SD). Differences in CO were analyzed using a student's t-test when normally distributed and a Wilcoxon test when abnormally distributed. A *P *< 0.05 was considered indicative of an absence of a type 1 error. For each patient, basic agreement between NICOM™, Vigileo™, and PAC-CCO was assessed using the ICC ratio and the Pearson correlation coefficient (r). The bias and the variability of the differences (NICOM™ vs. PAC-CCO, Vigileo™ vs. PAC-CCO and Vigileo™ vs. NICOM™) were illustrated using the modified Bland and Altman approach [7]. This traditional approach did not allow for all our predetermined criteria of clinical acceptability to be studied. In particular, precision is affected by natural CO changes, by the large variability, and the low time responsiveness of PAC-CCO [8]. To address these issues, we used the process developed previously [5]. Basically, we distinguished periods of stable, increasing, and decreasing CO using PAC-CCO slopes for optimal analysis of our criteria of clinical acceptability. We also studied periods of time where the application of standard protocols led to a hemodynamic challenge. Negative challenges were created when a lung recruitment test was performed and positive challenges were created by rapid fluid infusion and passive leg rising. Finally, the potential influence of systolic arterial pressure, pulmonary systolic artery pressure, and hematocrit (all factors known to potentially influence these methods) by assessing the bias and relative error of these three variables.

## Results

We studied 29 patients (26 men and 3 women), with a mean age of 63.2 ± 10.7 years, and a mean SAPS II of 36 ± 10. Surgical procedures consisted of 12 valves replacements, 12 coronary grafts, and 5 mixed-procedure operations. All patients were under mechanical ventilation at the start of the protocol, five patients received inotropic support, three received vasopressors, and five patients received vasodilators. Continuous recording of CO data was performed over 1210 minutes per patient (ranged from 1013 to 1454 minutes), allowing 12,099 simultaneous measurements to be obtained for each device (417 ± 107 per patient). PAC-CCO measurements, considered as the reference values, ranged from 2.10 to 12.80 L/minute (mean 4.86 ± 1.13 L/minute; Table 1). Comparison results between the three methods are displayed in Table 1 for global results.

**Table 1:** Comparison between the three methods for all periods (global results)

	Maximum	Minimum	Mean	SD
**CO value**				
PAC-CCO	12.8	2.1	4.9	1.8
NICOM™	13.1	1.4	4.8*†	1.1
Vigileo™	13.8	1.0	5.0	1.3
**Differences in CO**				
Vigileo™ – PAC-CCO	8.9	-7.8	-0.1	1.2
NICOM™ – PAC-CCO	6.8	-6.3	0.1**†**	1.5
NICOM™ – Vigileo™	8.6	-9.1	-0.1	1.8
**Relative errors**				
Vigileo™	1.97	0.0	0.19	0.17
NICOM™	1.37	0.0	0.23**†**	0.18

Relative error = (tested CO – PAC-CCO)/PAC-CCO.

* *P *< 0.05 vs. PAC-CCO, **† ***P *< 0.05 for NICOM™ vs. Vigileo™

CO = cardiac output; SD = standard deviation.

### Accuracy and precision

**Figure 1:**
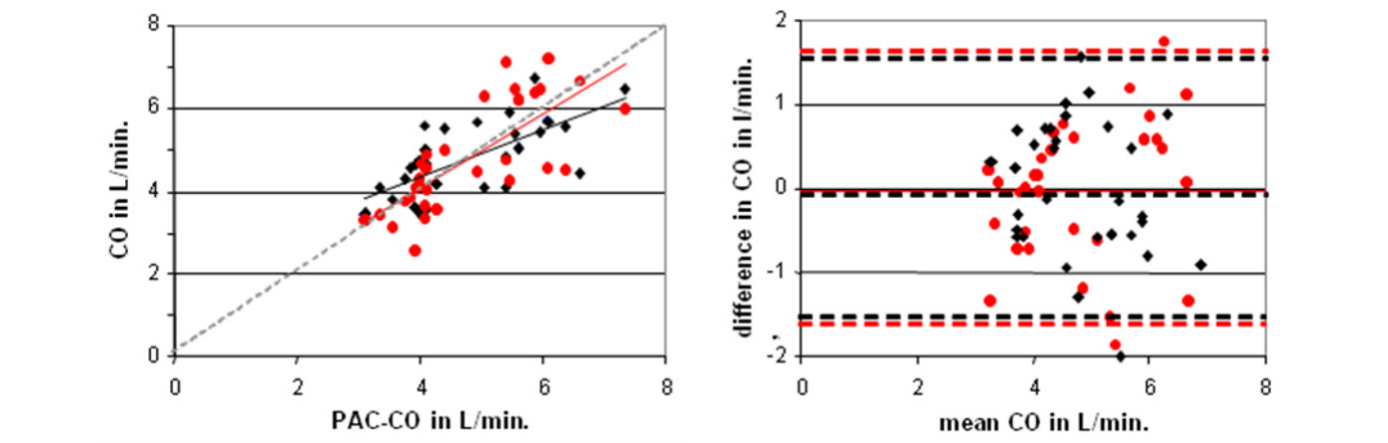
Comparison between NICOM™ and Vigileo™.  **(Left panel) **Relationship between averaged values of NICOM™ (in red, r = 0.77, not significant (NS) from identity line) and Vigileo™ (in black, r = 0,69, *P *< 0.05 from identity line) with PAC-CCO during periods of very stable cardiac output (CO). **(Right panel) **Corresponding Bland and Altman representation: NICOM™ bias = -0.01 L/min with limits of agreements (2 standard deviations) = 1.68 L/min; Vigileo™ bias = -0.01 L/min with limits of agreements (2 standard deviations) = 1.62 L/min.

After selection, 33 periods of very stable CO (PAC-CCO slope > 10%, SD/mean < 20%, representing 4133 points and 34% of the database) were specifically analyzed in order to minimize the effect of natural intra-patient CO variability and to determine optimally bias and precision. Results are summarized in Table 2. When all very stable CO values were averaged for each patient, correlations of NICOM™ and Vigileo™ with PAC-CCO were 0.77 and 0.69, respectively (Figure 1). The NICOM™ relative error was less than 30% in 94% of the patients and less than 20% in 79%. The corresponding ratios of the Vigileo™ were 91% and 79%, respectively (NS). The variability of measurements around the trend line (precision) was not different between the three methods: 8 ± 3%, 8 ± 4%, and 8 ± 3% for PAC-CCO, NICOM™, and Vigileo™, respectively. In all cases, the variability around the trend line was less than 20%. The bias of Vigileo™ was related to systolic pressure (r^2 ^= 0.19, *P *< 0.0001; Table 3). The bias of NICOM™ was marginally related to pulmonary systolic arterial pressure (r^2 ^= 0.009, *P *< 0.04) and haemoglobin blood level (r^2 ^= 0.04, *P *< 0.001; Tables 4 and 5). We did not find any other relationship between bias and any other factor.

**Table 2:** Comparison between the three methods restricted to the very stable period

	Maximum	Minimum	Mean	SD
**CO values**				
PAC-CCO	8.7	2.5	4.8	1.4
NICOM™	10.2	2.1	4.8	1.4
FloTrac-Vigileo™	11.2	1.0	5.0*	1.2
**Differences in CO**				
Vigileo™ – PAC-CCO	4.8	-3.0	0.1	0.9
NICOM™ – PAC-CCO	5.3	-2.7	0.0**†**	1.0
NICOM™ – Vigileo™	4.1	-5.1	-0.1	1.2
**Relative errors**				
Vigileo™	0.97	0.0	0.16	0.12
NICOM™	1.15	0.0	0.17**†**	0.12

Relative error = (tested CO – PAC-CCO)/PAC-CCO.

* *P *< 0.05 vs. PAC-CCO, **† ***P *< 0.05 for NICOM™ vs. Vigileo™

CO = cardiac output; SD = standard deviation.

**Table 3:** Impact of systolic arterial pressure level on Vigileo™ accuracy

Systolic blood pressure	Bias	Relative error
> 160 mmHg	2.2 ± 1.5 L/min	51 ± 36%
120 to 160 mmHg	0.6 ± 1.2 L/min.	15 ± 27%
80 to 120 mmHg	-0.1 ± 1.0 L/min	-0.0 ± 21%
< 80 mmHg	-0.9 ± 1.3 L/min	-18 ± 25%

**Table 4:** Impact of pulmonary systolic arterial pressure level on NICOM™ accuracy

Pulmonary pressure	Bias	Relative error
> 50 mmHg	0.5 ± 1.3 L/min	12 ± 31%
40 to 50 mmHg,	0.4 ± 1.4 L/min	11 ± 30%
< 50 mmHg,	0.1 ± 1.5 L/min	4 ± 30%

**Table 5:** Impact of hemoglobin blood level on NICOM™ accuracy

Hemoglobin (gr/L)	Bias	Relative error
> 140	0.0 ± 1.0 L/min	1 ± 24%
100 to 140	0.3 ± 1.5 L/min	7 ± 30%
< 100	-0.3 ± 1.4 L/min	-3 ± 28%

### Responsiveness

During acute hemodynamic challenges (19 patients; Table 6), CO changes were 7.2 minutes faster with Vigileo™ and 6.9 minutes faster with NICOM™ (*P *< 0.05 both for NICOM™ and Vigileo™ vs. PAC-CCO; NS for NICOM™ vs. Vigileo™). Amplitude of changes was not significantly different than thermodilution (Table 6).

**Table 6:** Responsiveness (time in minutes and amplitude in L) in 19 patients for which a hemodynamic challenge was performed

	Time			Amplitude		
	
	PAC-CCO	NICOM™	Vigileo™	PAC-CCO	NICOM™	Vigileo™
Negative	7.0 ± 2.6	1.3 ± 0.5*	1.1 ± 0.3*	-2.9 ± 1.0	-2.3 ± 0.8	-1.8 ± 0.6
Positive	9.4 ± 4.9	1.4 ± 0.5*	1.1 ± 0.3*	2.0 ± 1.0	2.4 ± 1.4	1.8 ± 1.0

* *P *< 0.05

Negative challenges correspond to expected decrease in cardiac output and conversely for positive challenges.

### Ability for detecting significant CO changes

We identified 37 periods of stable CO (PAC-CCO slope within +/- 10% representing 39% of the database); averaged slopes were 0.01 ± 0.06 for PAC-CCO, 0.03 ± 0.16 for NICOM™, and 0.00 ± 0.17 for Vigileo™ (NS for all comparisons). Unacceptable differences in slope compared with the reference were observed in two patients (5%) with the NICOM™ and three patients with the Vigileo™ (8%).

During 33 periods of increasing CO (PAC slope > 10%, 29% of the database), averaged slopes were 0.29 ± 0.21 for PAC-CCO, 0.30 ± 0.24 for NICOM™, and 0.15 ± 0.20 for Vigileo™ (*P *< 0.05 for Vigileo™ vs. other). Unacceptable differences in slope with the reference was observed in two cases (6%) with the NICOM™ and four patients with the Vigileo™ (12%).

During 31 periods of decreasing CO (PAC-CCO slope < 10%, 31% of the database), averaged slopes were -0.29 ± 0.18 for PAC-CCO, 0.21 ± 0.26 for NICOM™, and 0.20 ± 0.31 for Vigileo™ (NS for all) An unacceptable difference of slope with the reference was observed in four cases (13%) with the NICOM™ and five patients with the Vigileo™ (16%).

Finally, the sensitivity and specificity for predicting significant CO changes were 0.91 and 0.95, respectively, for the NICOM™ and 0.86 and 0.92, respectively, for the Vigileo™.

## Discussion

Although assessment of oxygen consumption (VO_2_) is limited by numerous difficulties, rapid adaptation of VO_2 _to metabolic needs remains conceptually one of the major objectives of hemodynamic resuscitation [9-11]. By neglecting soluble blood gases and considering the hemoglobin blood level as normal and stable, VO_2 _is a direct function of only three variables: cardiac output (CO), arterial oxygen saturation (SaO_2_), and mixed venous oxygen saturation (SvO_2_). The goal of hemodynamic care can therefore be schematically described as reaching and maintaining a specific combination of SaO_2_, SvO_2_, and CO values to meet estimated metabolic needs. Continuous and accurate monitoring of these three variables is consequently of major interest for early detection of acute events in any patient with or at risk for a compromised hemodynamic situation.

This study shows that a totally non-invasive method of CO monitoring can have the same performance as a moderately invasive tool in postoperative high-risk patients. Our results identify a negligible bias that is acceptable in 79% of individual cases for both NICOM™ and Vigileo™ according to our own restrictive tolerance criteria [5]. We considered a ± 20% tolerance because it is approximately the variability of the reference method [12-14]. Critchley and Critchley suggested that a ± 30% limit of agreement was acceptable for CO measurements [15]. However, this recommendation is based on limits of agreements, on a central value from the average of the two-tested technologies assuming that none of them is considered as a reference. Then, the real difference between the two-tested technologies may be more than 30%. Taking into consideration this level of tolerance assumes that PAC-CCO is not a reference and increases the accuracy of the Vigileo™ and NICOM™ systems to 91% and 94%, respectively.

Even if controversial, PAC-CCO was taken as reference because it remains the most widely used device for continuous CO monitoring in many settings [8,16-18]. Fick [19] or bolus thermodilution methods [14] could be considered as more robust references for CO snapshot measurements but we were interested in comparing these new automatic and continuous monitoring tools with a real equivalent monitoring reference. Using Fick or bolus thermodilution, it would have been impossible to compare precision and responsiveness because they require too much time due to manual data acquisition and averaging of several measurements. In addition, we considered PAC-CCO as a reference for accuracy when the PAC-CCO trend line slope was nearly flat and when the fluctuation of measurements around this trend line slope was small, indicating periods of CO stability. In such circumstances, the standard error of the mean (SEM) is given by the formula SEM = SD/√n. Even if PAC-CCO SD was 20%, the SEM was 2% when 100 points are averaged during a period of stable CO [20]. Then the lack of precision of the PAC-CCO can be compensated by the time during which stable CO values are averaged.

When studying a monitoring tool, precision and responsiveness may be of greater clinical importance than accuracy. The precision of both Vigileo™ and NICOM™, the two-tested devices, was similar and always clinically acceptable. The responsiveness of both devices was faster than continuous thermodilution and the amplitude responsiveness was not significantly different. Finally, sensitivity and specificity for detecting clinically relevant CO changes were good for both NICOM™ and Vigileo™ by comparison with PAC-CCO.

A significant difference with the PAC-CCO trend line slope was found in 5 to 13% of the cases for the NICOM™ and in 0 to 17% for the Vigileo™. In 21% of the patients, the bias was more than 20% for both NICOM™ and Vigileo™. It is obvious that several factors have artificially increased these proportions of unacceptable response. First, even when using the 'STAT' button (that provides a quicker re-assessment of CO), the PAC-CCO value is not really the averaged of one minute of measurement but takes into considerations the past five minutes. It results in a smoothing of acute CO changes and could have impacted our results. Second, even when CO is globally stable, the lag-time difference between NICOM™, Vigileo™, and thermodilution may have created transient disagreements in the minute-to-minute comparison. Third, results of the NICOM™ and Vigileo™ are more likely to be transiently altered by artifacts resulting from nurses' interventions and/or from patient movements. Fourth, the software that was included in the Vigileo™ device used in this study was the 1.01 version; this software should be upgraded in the future, leading to a potential improvement in its performances that will obviously require further clinical assessment. Finally, our study was performed on a selected population of postoperative cardiac patients. Our results cannot be translated to a wider range of clinical CO values, especially in high CO values and hyperdynamic states such as sepsis.

## Conclusions

In this study, the clinical acceptability of CO monitoring using a completely noninvasive technique (NICOM™) was equivalent to the performance of a minimally invasive technique (Vigileo™). The data was collected from postoperative cardiac surgery patients, limited by the high proportion of males to females, but included a wide range of CO values. According to our predetermined criteria, accuracy was acceptable in a large proportion of patients, precision was always clinically acceptable, and responsiveness was faster than thermodilution. We believe that the noninvasive bioreactance technology, considering its performance, should be added to the array of CO monitoring tools in selected patients.

## Key messages

• NICOM™, a noninvasive cardiac output monitoring system based on chest bioreactance, is equivalent to FloTrac-Vigileo™ in terms of accuracy, precision, time, and amplitude responsiveness.

## Abbreviations

CO: cardiac output; FloTrac-Vigileo™: cardiac output monitoring system based on arterial pressure signal; ICC: Intra Class Correlation; ICU: intensive care unit; NICOM™: Non Invasive Cardiac Output Monitoring system based on chest bioreactance; NS: not significant; PAC-CCO: continuous thermodilution using a pulmonary artery catheter; r: Pearson correlation coefficient; SaO_2_: arterial oxygen saturation; SAPS: Simplified Acute Physiologic Score; SD: standard deviation; SEM: standard error of the mean; SvO_2_: mixed venous oxygen saturation; VO_2_: oxygen consumption.

## Competing interests

AC, JCD, and PS are consultants for Edwards life Sciences. JDC and PS are members of the scientific advisory board of Cheetah-med.

## Authors' contributions

SM collected the data and drafted the manuscript. AC, JCD, and PS conceived of the study, performed the statistical analysis, and collaborated to finalize the manuscript. All authors read and approved the final manuscript.

## References

[B1] Spohr F, Hettrich P, Bauer H, Haas U, Martin E, Bottiger BW (2007). Comparison of two methods for enhanced continuous circulatory monitoring in patients with septic shock. Intensive Care Med.

[B2] de Wilde RB, Schreuder JJ, Berg PC van den, Jansen JR (2007). An evaluation of cardiac output by five arterial pulse contour techniques during cardiac surgery. Anaesthesia.

[B3] de Waal EE, Kalkman CJ, Rex S, Buhre WF (2007). Validation of a new arterial pulse contour-based cardiac output device. Crit Care Med.

[B4] Keren H, Burkhoff D, Squara P (2007). Evaluation of a noninvasive continuous cardiac output monitoring system based on thoracic bioreactance. Am J Physiol Heart Circ Physiol.

[B5] Squara P, Denjean D, Estagnasie P, Brusset A, Dib JC, Dubois C (2007). Noninvasive cardiac output monitoring (NICOM): a clinical validation. Intensive Care Med.

[B6] Squara P, Vincent JL (2008). Bioreactance: A new method for cardiac output monitoring. Year book of Intensive Care Medicine.

[B7] Bland JM, Altman DG (1986). Statistical methods for assessing agreement between two methods of clinical measurement. Lancet.

[B8] Haller M, Zollner C, Briegel J, Forst H (1995). Evaluation of a new continuous thermodilution cardiac output monitor in critically ill patients: a prospective criterion standard study. Crit Care Med.

[B9] Russell JA, Phang PT (1994). The oxygen delivery/consumption controversy. Approaches to management of the critically ill. Am J Respir Crit Care Med.

[B10] Squara P (2004). Matching total body oxygen consumption and delivery: a crucial objective?. Intensive Care Med.

[B11] Vermeij CG, Feenstra BW, Bruining HA (1990). Oxygen delivery and oxygen uptake in postoperative and septic patients. Chest.

[B12] Hillis LD, Firth BG, Winniford MD (1985). Analysis of factors affecting the variability of Fick versus indicator dilution measurements of cardiac output. Am J Cardiol.

[B13] Rubini A, Del Monte D, Catena V, Attar I, Cesaro M, Soranzo D, Rattazzi G, Alati GL (1995). Cardiac output measurement by the thermodilution method: an in vitro test of accuracy of three commercially available automatic cardiac output computers. Intensive Care Med.

[B14] Le Tulzo Y, Belghith M, Seguin P, Dall'Ava J, Monchi M, Thomas R, Dhainaut JF (1996). Reproducibility of thermodilution cardiac output determination in critically ill patients: comparison between bolus and continuous method. J Clin Monit.

[B15] Critchley LA, Critchley JA (1999). A meta-analysis of studies using bias and precision statistics to compare cardiac output measurement techniques. J Clin Monit Comput.

[B16] Boldt J, Menges T, Wollbruck M, Hammermann H, Hempelmann G (1994). Is continuous cardiac output measurement using thermodilution reliable in the critically ill patient?. Crit Care Med.

[B17] Nelson LD (1997). The new pulmonary artery catheters: continuous venous oximetry, right ventricular ejection fraction, and continuous cardiac output. New Horiz.

[B18] Mihm FG, Gettinger A, Hanson CW, Gilbert HC, Stover EP, Vender JS, Beerle B, Haddow G (1998). A multicenter evaluation of a new continuous cardiac output pulmonary artery catheter system. Crit Care Med.

[B19] Stetz CW, Miller RG, Kelly GE, Raffin TA (1982). Reliability of the thermodilution method in the determination of cardiac output in clinical practice. Am Rev Respir Dis.

[B20] Cecconi M, Dawson D, Grounds R, Rhodes A (2008). Lithium dilution cardiac output measurement in the critically ill patient: determination of precision of the technique. Intensive Care Med.

